# Synthesis and Cytotoxic Effect of Some Novel 1,2-Dihydropyridin-3-carbonitrile and Nicotinonitrile Derivatives

**DOI:** 10.3390/molecules21010030

**Published:** 2015-12-31

**Authors:** Eman M. Flefel, Hebat-Allah S. Abbas, Randa E. Abdel Mageid, Wafaa A. Zaghary

**Affiliations:** 1 Department of Chemistry, College of Science, Taibah University, Al-Madinah Al-Monawarah 1343, Saudi Arabia; emanmflefel@yahoo.com; 2 Department of Photochemistry, National Research Centre, Dokki, Cairo 12622, Egypt; randaabdelmagid@yahoo.com; 3 Department of Chemistry, College of Science, King Khalid University, Abha 9004, Saudi Arabia; 4 Department of Pharmaceutical Chemistry, College of Pharmacy, Helwan University, Ain Helwan, Cairo 11795, Egypt; wzaghary@yahoo.com

**Keywords:** nicotinonitrile, 4-fluorophenylpyridine, acetohydrazide, chloropyridine, cytotoxicity

## Abstract

1-(2,4-Dichlorophenyl)-3-(4-fluorophenyl)propen-1-one (**1**) was prepared and reacted with an active methylene compound (ethyl cyanoacetate) in the presence of ammonium acetate to give the corresponding cyanopyridone **2**. Compound **2** reacted with hydrazine hydrate, malononitrile, ethyl bromoacetate and phosphorous oxychloride to afford compounds **4** and **7**–**11**, respectively. The 2-chloropyridine derivative **11** reacted with different primary amines, namely benzyl amine, piperonyl amine, 1-phenylethyl amine, and/or the secondary amines 2-methyl-pipridine and morpholine to give the corresponding derivatives **12**–**15**. Hydrazinolysis of chloropyridine derivative **11** with hydrazine hydrate afforded the corresponding hydrazino derivative **17**. Condensation of compound **17** with ethyl acetoacetate, acetylacetone, isatin and different aldehydes gave the corresponding derivatives **18**–**21**. Some of newly synthesized compounds were screened for cytotoxic activity against three tumor cell lines. The results indicated that compounds **8** and **16** showed the best results, exhibiting the highest inhibitory effects towards the three tumor cell lines, which were higher than that of the reference doxorubicin and these compounds were non-cytotoxic towards normal cells (IC_50_ values > 100 μg/mL).

## 1. Introduction

Cancer is the second leading cause of death in both developing and developed countries [1]. The leading forms were lung cancer, colorectal cancer, liver cancer and breast cancer [2,3]. Cancer treatment has been a major research and development effort in academia and the pharmaceutical industry for numerous years [4,5]. Despite the fact that there is a large amount of information available dealing with the clinical aspects of cancer chemotherapy, we felt that there was a clear requirement for an updated treatment from the point of view of medicinal chemistry and drug design [6]. Another major goal for developing new anticancer agents is to overcome cancer resistance to drug treatment, which has made many of the currently available chemotherapeutic agents ineffective [7].

**Figure 1:**
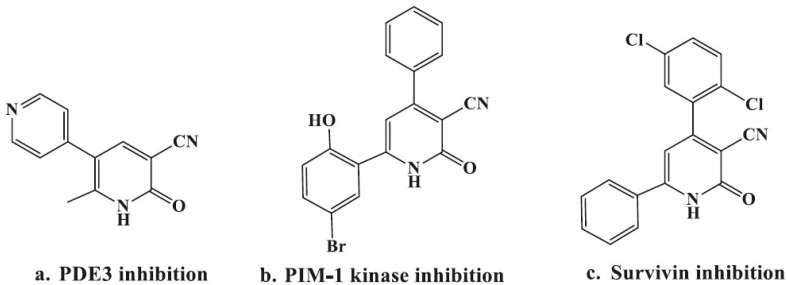
Various 3-cyano-2-oxopyridine derivatives with potential growth inhibitory and/or antiangiogenic actions through PDE3 inhibition (**a**); PIM-1 kinase inhibition (**b**); or survivin inhibition (**c**).

Chalcones, one of the major classes of natural products with widespread occurrence in vegetables, fruits, spices and soy-based foodstuffs, have been reported to possess several biological activities such as antibacterial [8,9], anti-fungal [10,11], anti-inflammatory [12], and anti-tumor activities [13,14]. An important feature of chalcones is their ability to act as an intermediate for the synthesis of biologically active heterocyclic compounds such as pyrimidine and pyridine derivatives [15,16]. The pyridine nucleus is an integral part of anti-inflammatory and anticancer agents [17,18]. On the other hand, cyanopyridone and cyanopyridine derivatives have shown to possess promising antimicrobial [19] antioxidant [20,21], antibiotic [22], antiinflamatory [23,24], analgesic, anticonvulsant [25] and anticancer [26,27,28,29] properties. 3-Cyano-2-pyridones are analogous to the alkaloid ricinine, the first known alkaloid containing a cyano group. The anticancer activity of 3-cyano-2-pyridone derivatives is of much interest owing to the different types of biological targets they might interfere with, e.g., PDE3, PIM1 kinase, and survivin (Figure 1) [30].

Motivated by the above recent literature observations and our own previous reports [20,21,31,32,33], herein some new pyridine derivatives were synthesized, leading to interesting heterocyclic scaffolds that are mostly useful for the creation of varied chemical libraries of drug-like molecules for biological screening.

## 2. Results and Discussion

### 2.1. Chemistry

**Scheme 1:**
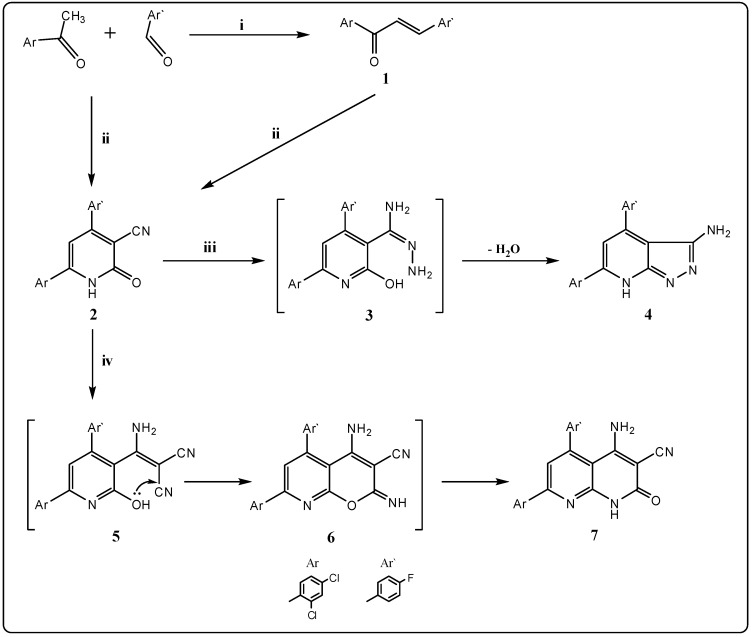
General methods for the preparation of compounds **2**–**7**. Reagents and conditions: (**i**) NaOH/EtOH, stirring; (**ii**) ethyl cyanoacetate/CH_3_COONH_4_/EtOH, reflux; (**iii**) hydrazine hydrate 98% (1 mL)/EtOH, reflux; and (**iv**) malononitrile/triethylamine (3 mL)/EtOH, reflux.

**Scheme 2:**
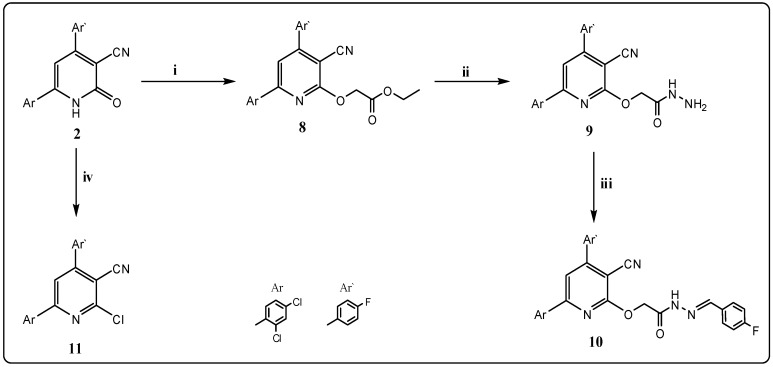
General methods for the preparation of compounds **8**–**11**. Reagents and conditions: (**i**) ethyl bromoacetate/anh. K_2_CO_3_/dry CH_3_COCH_3_, reflux; (**ii**) hydrazine hydrate 98% (2 mL)/EtOH, reflux; (**iii**) 4-flurobenzaldehyde/EtOH, reflux; and (**iv**) phosphorus oxychloride/EtOH, reflux.

**Scheme 3:**
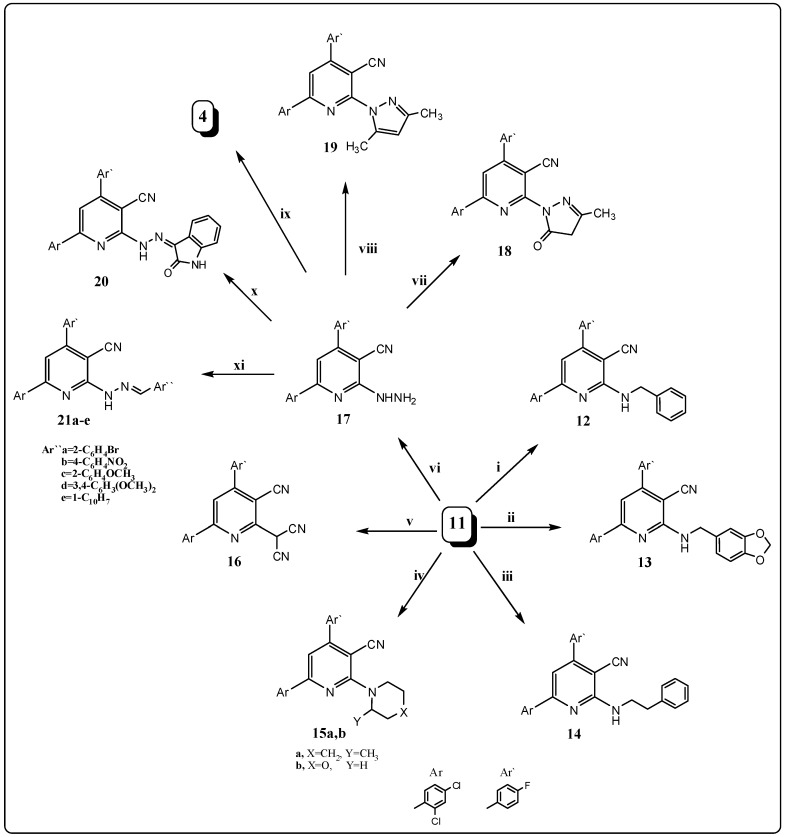
General methods for the preparation of compounds **12**–**21a**–**e**. Reagents and conditions: (**i**) benzylamine/EtOH, reflux; (**ii**) piperonylamine/EtOH, reflux; (**iii**) 1-phenylethylamine/EtOH, reflux; (**iv**) 2-methylpiperidine or morpholine/EtOH, reflux; (**v**) malononitrile/triethylamine (1 mL)/EtOH, reflux; (**vi**) hydrazine hydrate 98% (2 mL)/EtOH, reflux; (**vii**) ethyl acetoacetate/AcOH, reflux; (**viii**) acetylacetone/AcOH, reflux; (**ix**) DMF or AcOH, reflux; (**x**) isatin/3 drops AcOH/EtOH, reflux; and (**xi**) appropriate aromatic aldehyde, namely: 2-bromobenzaldhyde, 4-nitrobenzaldhyde, 2-methoxy-benzaldhyde, 3,4-dimethoxybenzaldhyde and/or1-naphthaldehyde/3 drops AcOH/EtOH, reflux.

The synthesis of the designed target compounds was achieved as outlined in Scheme 1, Scheme 2 and Scheme 3. During this investigation, the pyridin-3-carbonitrile starting material **2** was prepared by condensation of the corresponding enone **1** [34] with ethyl cyanoacetate in the presence of excess ammonium acetate (Scheme 1). Compound **2** can also be obtained in high yield through a four-component modified Hantzch reaction, in a one-step synthesis, by refluxing a mixture of 2,4-dichloro-acetophenone, 4-fluorobenzaldehyde, ethyl cyanoacetate and ammonium acetate in *n*-butanol. The structure of pyridin-3-carbonitrile **2** was supported by elemental analysis, IR, (^1^H, ^13^C) NMR and mass spectral studies. Its IR spectrum showed absorption bands at 3278, 2219, 1632 cm^−1^ indicating the presence of NH, CN and CO groups, respectively. Its ^1^H-NMR spectrum displayed a broad D_2_O exchangeable singlet at δ 8.10 ppm for the NH proton, while its ^13^C-NMR spectrum also revealed signals at δ 117.6 and 161.8 ppm for CN and CO moieties, respectively. The mass spectrum showed a molecular ion peak at *m*/*z* 358 (M^+^, 98%), which tallies with its molecular formula C_18_H_9_Cl_2_FN_2_O.

Pyridin-3-carbonitrile **2** possesses several reactive sites, *viz*. CN, NH, and CO groups, which can play a great role in the synthesis of heterocyclic derivatives, most of which are interesting from both the chemical and biological point of view. Thus, hydrazinolysis of pyridin-3-carbonitrile **2** with hydrazine hydrate in absolute ethanol for 15 h affords the corresponding pyrazolo[3,4-*b*]pyridin-3-amine derivative **4** through the elimination of a water molecule from the intermediate **3** (Scheme 1). Pyrazolo[3,4-*b*]pyridine derivative **4** was identified by the absence of the cyano and carbonyl groups signals in its IR and the presence of an amino group signal at δ 5.69 ppm and the broad band of the NH proton at δ 10.05 ppm in its ^1^H-NMR spectrum. Its mass spectrum showed a molecular ion peak at *m*/*z* 372 (M^+^; 72%), which conforms to its molecular formula C_18_H_11_Cl_2_FN_4_.

Compound **2** was also refluxed with malononitrile to afford 4-amino-7-(2,4-dichlorophenyl)-5-(4-fluorophenyl)-2-oxo-1,2-dihydro-1,8-naphthyridine-3-carbonitrile (**7**) via the intermediates **5** and **6**, as confirmed by elemental analysis, ^1^H- and ^13^C-NMR. The R spectrum of compound **7** showed bands at 3312, 3249, 3145 and 1688 cm^−1^ due to NH_2_, NH and CO groups, respectively; its ^13^C-NMR spectrum showed signals at δ 118.1 and 168.8 ppm corresponding to CN and CO groups, respectively. Its mass spectrum showed a molecular ion peak at *m*/*z* 424 (M^+^; 92%), which conforms to its molecular formula C_21_H_11_Cl_2_FN_4_O.

Moreover, when pyridin-3-carbonitrile **2** was alkylated with ethyl bromoacetate in acetone using anhydrous potassium carbonate as catalyst, the ester derivatives **8** was produced (Scheme 2). The ^1^H-NMR spectrum of **8** showed signals at δ 1.15, 4.13 and 5.11 ppm due to the presence of (C*H*_3_-ester), (OC*H*_2_-ester) and (O–C*H*_2_) respectively; and its ^13^C-NMR exhibited signals at δ 13.5, 43.8, 61.3 and 167.9 ppm due to (*C*H_3_), (2*C*H_2_) and (CO) groups, respectively.

Ester derivative **8** were condensed with hydrazine hydrate (98%) in ethanol to give 2-[3-cyano-6-(2,4-dichlorophenyl)-4-(4-fluorophenyl)pyridin-2-yloxy]acetohydrazide (**9**) (Scheme 2), confirmed by its IR and NMR spectra. Its IR spectrum showed strong peaks at 3314, 3282 and 3116 cm^−1^ indicating the presence of a –NHNH_2_ group, and the NMR (^1^H and ^13^C) and mass spectra were also in accordance with its structure. 

Schiff base **10** can be produced via condensation of acetohydrazide **9** with an aromatic aldehyde, namely 4-flourobenzaldehyde, in ethanol (Scheme 2). The structure of Schiff base **10** was elucidated based on the spectral and analytical data. The IR spectrum revealed the absence of the absorption bands of (NH_2_) group absorption and its ^1^H-NMR spectrum showed a singlet at δ 8.10 ppm due to the presence of the (C*H*=N–) group.

In addition, chlorination of cyanopyridone **2** with phosphorous oxychloride afforded the 2-chloronicotinonitrile derivative **11** (Scheme 2) in good yield, after 8 h. The IR spectrum showed the absence of a characteristic CO group band.

It is known that position 2 in chloronicotinonitrile derivatives shows distinct activities toward nucleophiles, especially nitrogen nucleophiles. Thus, nucleophilic replacement of the chlorine atom of chloronicotinonitrile **11** was performed by refluxing with different primary amines, namely benzyl- amine, piperonylamine, 1-phenylethylamine and/or secondary amines, namely 2-methyl-piperidine and morpholine in boiling ethanol for 6–12 h to afford the corresponding 2-aminopyridine derivatives **12**–**15a**,**b**; respectively (Scheme 3). The elemental analysis and spectral data of compounds **12**–**15a**,**b** were in agreement with the proposed structures. The ^1^H-NMR of compound **15b** for example, showed signals at δ 3.31 and 3.72 ppm due to the presence of (2N–C*H*_2_) and (2O–C*H*_2_) groups, respectively; and its ^13^C-NMR exhibited signals at δ 47.9, 49.1, 64.10 and 65.9 ppm due to the presence of (2N–*C*H_2_) and (2O–*C*H_2_), respectively.

Furthermore, nucleophilic displacement was carried out by heating the chloropyridine derivative **11** with malononitrile in ethanol containing a few drops of triethylamine as a catalyst to give 2-[3-cyano-6-(2,4-dichlorophenyl)-4-(4-fluorophenyl)pyridin-2-yl]malononitrile (**16**). The structure of compound **16** was confirmed by its spectral data; the IR spectrum showed the presence of the CN group at 2218, 2225 cm^−1^. In addition, the NMR (^1^H and ^13^C) and mass spectral data were in accordance with its structure. Hydrazinolysis of the chloropyridine derivative **11** was performed by its reaction with excess hydrazine hydrate in refluxing ethanol to give the hydrazino derivative **17** (Scheme 3). The structure of **17** was confirmed by its spectral data. The IR spectrum exhibited the characteristic absorption bands at 3440, 3320, 3150 cm^−1^ indicating the presence of the –NHNH_2_ group. Its mass spectrum showed a molecular ion peak at *m*/*z* 372 (M^+^; 39%), which conforms to its molecular formula C_18_H_11_Cl_2_FN_4_.

The 2-hydrazino-nicotinonitrile **17** is another key compound, which facilitates the synthesis of diverse heterocyclic compounds. Thus, it reacted with different active methylene (β-diketones), namely: ethyl acetoacetate and acetylacetone in glacial acetic acid, and thus the *N*-pyrazolo derivatives **18** and **19** were produced (Scheme 3). The IR spectrum of compound **19,** for example, showed a characteristic band at 2210 cm^−1^ for the CN group and its ^1^H-NMR spectrum revealed singlets at δ 2.31, 2.45 and 6.15 ppm due to (2C*H*_3_) and the (C*H*-pyrazole) moieties, respectively. The ^13^C-NMR data displayed two characteristic signals at δ 18.4, 19.3 and 117.9 ppm for 2*C*H_3_ and CN groups, respectively. Also, on heating compound **17** with isatin in ethanol it afforded 6-(2,4-dichlorophenyl)-4-(4-fluorophenyl)-2-(2-(2-oxoindolin-3-ylidene)hydrazinyl)nicotinonitrile (**20**) in good yield (Scheme 3). The structure of compound **20** gave correct elemental analyses values and spectral features.

In addition, to get a new series of Schiff bases expected to be biologically active, heating of 2-hydrazinonicotinonitrile **17** with different aromatic aldehydes, namely 2-bromobenzaldehyde, 4-nitrobenzaldeyde, 2-methoxybenzaldeyde, 3,4-dimethoxybenzaldeyde and/or 1-naphthaldeyde in ethanol gave the corresponding Schiff bases **21a**–**e**, respectively. The structure of compounds **21a**–**e** was characterized by the disappearance of the NH_2_ group. In addition, the ^1^H-NMR spectra showed a singlet at around δ 8.31–8.33 due to the presence of the azomethine group (C*H*=N–). Finally, reaction of 2-hydrazinonicotinonitrile **17** with acetic acid or DMF afforded the corresponding pyrazolo[3,4-*b*]pyridin-3-amine derivative **4** through intramolcular cyclization via the addition of the NH_2_ functional group at the CN group.

### 2.2. In Vitro Anticancer Screening

The *in vitro* cytotoxic activity the newly synthesized compounds against human breast cell line (MCF7), non-small cell lung cancer NCI-H460, CNS cancer SF-268 and WI 38 (normal fibroblast cells) were evaluated using doxorubicin as the reference drug, according to the method reported by Skehan *et al.* [35]. The IC_50_ values of the synthesized compounds compared to the reference drug are shown in Table 1.

**Table 1:** Cytotoxic activity in (IC_50_, μg/mL) by the newly synthesized compounds against human cancer cell lines and normal cells.

Comp. No.	IC_50_ (μg/mL)			
	MCF-7	NCI-H460	SF-268	WI 38
**4**	67.04 ± 6.23 ^c^	56.75 ± 8.20 ^c^	69.05 ± 9.15 ^c^	18.62 ± 1.21
**7**	36.22 ± 2.14 ^c^	74.03 ± 3.65 ^c^	62.13 ± 3.61 ^c^	22.97 ± 8.2
**8**	0.02 ± 0.002 ^a^	0.01 ± 0.002 ^a^	0.02 ± 0.045 ^a^	non-cytotoxic
**9**	2.41 ± 1.24 ^a^	2.30 ± 2.86 ^a^	0.46 ± 0.06 ^a^	62.19 ± 2.02
**10**	30.58 ± 1.10 ^b^	30.67 ± 1.64 ^b^	28.18 ± 8.83 ^b^	19.80 ± 2.68
**13**	16.26 ± 1.87 ^b^	18.92 ± 1.03 ^b^	23.24 ± 4.12 ^b^	20.38 ± 4.99
**15a**	37.07 ± 7.34 ^c^	16.37 ± 2.32 ^b^	38.94 ± 2.63 ^c^	30.62 ± 6.21
**16**	0.01 ± 0.002 ^a^	0.02 ± 0.001 ^a^	0.01 ± 0.003 ^a^	non-cytotoxic
**17**	0.61 ± 0.082 ^a^	0.86 ± 0.02 ^a^	2.19 ± 0.83 ^a^	64.11 ± 1.22
**18**	20.22 ± 2.26 ^b^	0.01 ± 0.003 ^a^	20.20 ± 3.26 ^b^	29.82 ± 4.88
**19**	75.20 ± 13.86 ^c^	62.30 ± 10.35 ^c^	10.39 ± 4.19 ^a^	50.20 ± 10.22
**20**	0.66 ± 0.21 ^a^	0.90 ± 0.12 ^a^	2.34 ± 0.51 ^a^	72.45 ± 2.40
**21d**	66.02 ± 8.25 ^c^	44.95 ± 10.46 ^c^	32.45 ± 6.04 ^b^	non-cytotoxic
DMSO	0	0	0	0
Doxorubicin	0.04 ± 0.008	0.09 ± 0.008	0.09 ± 0.007	non-cytotoxic

MCF-7 (breast adenocarcinoma); NCI-H460 (non-small cell lung cancer); SF-268 (CNS cancer); WI 38 (normal fibroblast cells); Doxorubicin (anticancer positive control); DMSO (solvent, negative control); ^a^ highly active; ^b^ moderately active; ^c^ weakly active.

**Figure 2:**
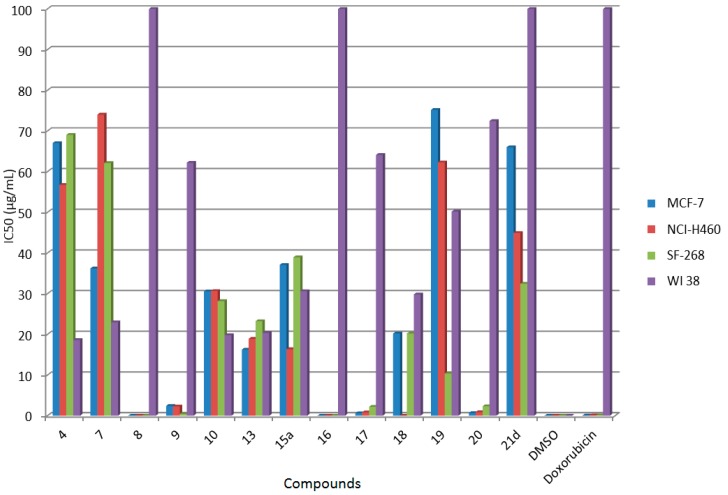
Cytotoxic activity of some newly synthesized compounds against human cancer cell lines and normal cells.

From the results presented in Table 1 and Figure 2, it is evident that some of the compounds were active against the three human cancer cell lines. Compounds **8** and **16** displayed high cytotoxic activity against the tested cell lines (most of the IC_50_ values ranged from 0.01 ± 0.002 to 0.02 ± 0.001 μg/mL) and these compounds were non-cytotoxic on the normal cells (IC_50_ values > 100 μg/mL) and exhibited better cytotoxicity against most of cancer cell lines than doxorubicin as standard drug. Moreover, compounds **9**, **17** and **20** exhibited high growth inhibitory activity on the various cancer panel cell lines (IC_50_ values ranged from 0.46 ± 0.006 to 2.43 ± 0.51 μg/mL) with weak cytotoxicity on the normal cells (IC_50_ values ranged from 62.19 ± 2.02 to 72.45 ± 2.40 μg/mL). In addition, other compounds showed moderate to weak cytotoxicity against all cancer cell lines (IC_50_ values ranged from 10.39 ± 4.19 to 75.20 ± 13.86 μg/mL) with cytotoxic effects on the human normal cell (IC_50_ values ranged from non-cytotoxic to 50.20 ± 10.22 μg/mL) in comparison with doxorubicin. The resultant data can be analyzed with respect to the chemical structures of the examined compounds; thus it can be noticed that the derivatives **8** and **16** that bear ester or malononitrile side chains on the parent cyanopyrine nucleus showed the highest potency as growth inhibiting agents against the three human cancer cell lines, which might be due to their lipophilicity that allows their accumulation inside tumor tissues inducing growth inhibition effects [36].

## 3. Experimental Section

### 3.1. General Information

All melting points are uncorrected and were determined on a Stuart electric melting point apparatus. The microanalyses were within ±0.4% of the theoretical values and were carried out at the Microanalytical Centre, National Research Centre, Cairo, Egypt. IR spectra (KBr) were recorded on a FT-IR 400D infrared spectrometer (Shizmadu-series, Kyoto, Japan) using the OMNIC program and are reported as frequency of absorption in cm^−1^. ^1^H-NMR spectra were recorded on a Bruker (Rheinstetten, Germany) spectrophotometer at 400 MHz using TMS as internal standard and with residual signals of the deuterated solvent δ = 7.26 ppm for CDCl_3_ and δ 2.51 ppm for DMSO-*d*_6_. ^13^C-NMR spectra were recorded on the same spectrometer at 100 MHz and referenced to solvent signals δ = 77 ppm for CDCl_3_ and δ 39.50 ppm for DMSO-*d*_6_. The mass spectra were recorded on a Shimadzu GCMS-QP-1000 EX mass spectrometer (Kyoto, Japan) at 70 eV using the electron ionization technique. Homogeneity of all compounds synthesized was checked by TLC which was performed on Merck 60 (Munich, Germany) ready-to-use silica gel plates to monitor the reactions and test the purity of the new synthesized compounds. The chemical names given for the prepared compounds are according to the IUPAC system.

### 3.2. Synthetic Procedures

#### 3.2.1. 6-(2,4-Dichlorophenyl)-4-(4-fluorophenyl)-2-oxo-1,2-dihydropyridin-3-carbonitrile (**2**)

*Method A*: A mixture of 1-(2,4-dichlorophenyl)-3-(4-fluorophenyl)prop-2-en-1-one (**1**, 2.95 g, 0.01 mol), ethyl cyanoacetate (1.13 g, 0.01 mol) and ammonium acetate (6.16 g, 0.08 mol) in ethanol (40 mL) was refluxed for 10 h. After cooling, the precipitate was filtered, dried and recrystallized from dioxane to give compound **2** (35% yield).

*Method B:* A mixture of 2,4-dichloroacetophenone (1.88 g, 0.01 mol), 4-fluorobenzaldehyde (1.24 g, 0.01 mol), ethyl cyanoacetate (1.13 g, 0.01 mol) and ammonium acetate (6.16 g, 0.08 mol) in *n*-butanol (20 mL) was refluxed for 3 h, to give yellow crystals that were then filtered, washed with water, dried and recrystallized to give the title compound **2** (85% yield).

*Compound* **2**: m.p. 276–277 °C; IR (KBr) ν_max_ in cm^−1^: 3278 (NH), 2219 (CN), 1632 (C=O); ^1^H-NMR (DMSO-*d*_6_): 6.61 (s, 1H, pyridine H5), 7.41–7.85 (m, 7H, Ar–H), 8.10 (br. s, 1H, NH, D_2_O exchangeable); ^13^C-NMR (DMSO-*d*_6_): 117.6 (CN), 119.61, 120.23, 122.00, 122.54, 123.33, 123.59, 133.93, 135.87, 136,12, 142.45, 144.64, 146.21, 146.87, 151,32, 151.98 (16 Ar–C), 161.8 (C=O); MS, *m*/*z* (%): 358 [M]^+^ (98), 360 [M + 2]^+^ (59), 362 (M^+^ + 4; 11%). Anal. Calcd. for C_18_H_9_Cl_2_FN_2_O (359.18): C, 60.19; H, 2.53; N, 7.80%; found: C, 59.97 ; H, 2.74; N, 7.94%.

#### 3.2.2. 6-(2,4-Dichlorophenyl)-4-(4-fluorophenyl)-7*H*-pyrazolo[3,4-*b*]pyridin-3-amine (**4**)

*Method A*: A mixture of compound **2** (3.6 g, 0.01 mol) and hydrazine hydrate 98% (1 mL, 0.02 mol) in absolute ethanol (30 mL) was refluxed for 15 h. The reaction mixture was left at room temperature overnight and then poured into ice/cold water to complete precipitation. The product was filtered, dried and recrystallized from benzene to give compound **4** (75% yield).

*Method B*: Compound **17** (3.7 g, 0.01 mol) in DMF or AcOH (20 mL) was refluxed for 8 h. The reaction mixture was cooled; the solid product that precipitated was filtered, dried and recrystallized from ethanol to give compound **4** (43% yield).

*Compound* **4**: m.p. 309–311 °C; IR (KBr) ν_max_ in cm^−1^: 3414, 3335, 3180 (NH_2_, NH); ^1^H-NMR (DMSO-*d*_6_): 5.69 (s, 2H, NH_2_, D_2_O exchangeable), 7.12–7.97 (m, 8H, Ar–H + pyridine H5), 10.05 (s, H, NH, D_2_O exchangeable); ^13^C-NMR (DMSO-*d*_6_): 109.76, 111.54, 112.32, 114.29, 115.21, 115.98, 120.54, 121.87, 123.34, 126.30, 132.64, 138.97, 144.54, 145.92, 148.40 (16 Ar–C), 157.1, 158.9 (2C=N); MS, *m*/*z* (%): 372 [M]^+^ (72), 374 [M + 2]^+^ (40), 376 [M + 4]^+^ (8). Anal. Calcd. for C_18_H_11_Cl_2_FN_4_ (373.21): C, 57.93; H, 2.97; N, 15.01%; found: C, 58.12 ; H, 2.70; N, 15.23%.

#### 3.2.3. 4-Amino-7-(2,4-dichlorophenyl)-5-(4-fluorophenyl)-2-oxo-1,2-dihydro-1,8-naphthyridine-3-carbonitrile (**7**)

To a solution of compound **2** (3.6 g, 0.01 mol) in absolute ethanol (30 mL), triethylamine (3 mL), and malononitrile (0.7 g, 0.01 mol) were added. The reaction mixture was refluxed for 6 h., then left to cool to room temperature, poured into cold water and neutralized with diluted hydrochloric acid to complete precipitation. The solid obtained was filtered, washed with water, dried and recrystallized from methanol to give compound **7**. Yield 70%; m.p. 188–189 °C; IR (KBr) ν_max_ in cm^−1^: 3312, 3249, 3145 (NH_2_, NH), 2223 (CN), 1688 (C=O); ^1^H-NMR (DMSO-*d*_6_): 5.46 (s, 2H, NH_2_, D_2_O exchangeable), 6.89 (s, 1H, pyridine H5), 7.10–7.41 (m, 7H, Ar–H), 8.20 (s, 1H, NH, D_2_O exchangeable); ^13^C-NMR (DMSO-*d*_6_): 118.1 (CN), 121.32, 122.87, 123.76, 125.32, 128.43, 132.65, 133.87, 134.12, 138.09, 139.56, 141.54, 147.65, 148.02, 149.53 (18 Ar–C), 158.6 (C=N), 168.9 (C=O); MS, *m*/*z* (%): 424 [M]^+^ (92), 426 [M + 2]^+^ (59), 428 [M + 4]^+^ (10). Anal. Calcd. for C_21_H_11_Cl_2_FN_4_O (425.24): C, 59.31; H, 2.61; N, 13.18%; found: C, 59.12; H, 2.79; N, 13.33%.

#### 3.2.4. Ethyl 2-[3-cyano-6-(2,4-dichlorophenyl)-4-(4-fluorophenyl)pyridin-2-yloxy]acetate (**8**)

A mixture of compound **2** (3.6 g, 0.01 mol), ethyl bromoacetate (1.2 mL, 0.01 mol) and anhydrous potassium carbonate (2.10 g, 0.015 mol) in dry acetone (50 mL) was refluxed for 24 h. The reaction mixture was cooled and poured onto ice/cold water; the solid that separated out was filtered, dried and recrystallized from dioxane to give compound **8**. Yield 75%; m.p. 119–121 °C; IR (KBr) ν_max_ in cm^−1^: 2220 (CN), 1755 (C=O ester); ^1^H-NMR (DMSO-*d*_6_): 1.15 (t, *J* = 7.5 Hz, 3H, CH_3_-ester), 4.13 (q, *J* = 7.5 Hz, 2H, O-C*H*_2_-ester), 5.11 (s, 2H, O-C*H*_2_), 7.00–7.40 (m, 8H, Ar–H + pyridine H5); ^13^C-NMR (DMSO-*d*_6_): 13.5 (CH_3_), 44.8, 61.3 (2CH_2_), 118.6 (CN), 123.30, 123.54, 127.10, 127.98, 131.34, 133.54, 137.07, 138.76, 141.99, 142.76, 144.65, 126.87, 148.43 (16 Ar–C), 159.1 (C=N), 167.9 (C=O); MS, *m*/*z* (%): 444 [M]^+^ (61), 446 [M + 2]^+^ (37), 448 [M + 4]^+^ (6), 371 [M–COOC_2_H_5_]^+^ (41). Anal. Calcd. for C_22_H_15_Cl_2_FN_2_O_3_ (445.27): C, 59.34; H, 3.40; N, 6.29%; found: C, 59.52 ; H, 3.71; N, 6.05%.

#### 3.2.5. 2-[3-Cyano-6-(2,4-dichlorophenyl)-4-(4-fluorophenyl)pyridin-2-yloxy]acetohydrazide (**9**)

A mixture of compound **8** (4.5 g, 0.01 mol), hydrazine hydrate 98% (2 mL, 0.04 mol) and absolute ethanol (30 mL) was refluxed for 4 h. The reaction mixture was cooled and the formed solid was filtered, dried and recrystallized from acetic acid to give compound **9**. Yield 76%; m.p. 197–199 °C; IR (KBr) ν_max_ in cm^−1^: 3314, 3282, 3116 (NH_2_, NH), 2225 (CN), 1696 (C=O ester); ^1^H-NMR (DMSO-*d*_6_): 4.92 (s, 2H, CH_2_), 7.00–7.40 (m, 8H, Ar–H + pyridine H5), 8.2 (s, 1H, NH, D_2_O exchangeable), 9.8 (s, 2H, NH_2_, D_2_O exchangeable); ^13^C-NMR (DMSO-*d*_6_): 62.4 (CH_2_), 119.7 (CN), 121.65, 124.05, 124,90, 125.71, 128.23, 129.98, 133.76, 137.54, 142.12, 143.45, 145.86 (16 Ar–C), 158.6 (C=N), 169.4 (C=O); MS, *m*/*z* (%): 430 [M]^+^ (31), 432 [M + 2]^+^ (20), 434 [M + 4]^+^ (4), 371 [M–CONHNH_2_]^+^ (8). Anal. Calcd. for C_20_H_13_Cl_2_FN_4_O_2_ (431.25): C, 55.70; H, 3.04; N, 12.99%; found: C, 55.52; H, 3.21; N, 13.05%.

#### 3.2.6. 2-[3-Cyano-6-(2,4-dichlorophenyl)-4-(4-fluorophenyl)pyridin-2-yloxy]-*N*′-(4-fluorobenzylidene)-acetohydrazide (**10**)

A mixture of compound **9** (4.3 g, 0.01 mol) and 4-fluorobenzaldehyde (1.24 g, 0.01 mol) in ethanol (20 mL) was refluxed for 6 h. The solid formed after cooling was filtered, dried and recrystallized from acetic acid to give compound **10**. Yield 79%; m.p. 219–221 °C ; IR (KBr) ν_max_ in cm^−1^: 3218 (NH), 2226 (CN), 1665 (C=O); ^1^H-NMR (DMSO-*d*_6_): 5.1 (s, 2H, O–CH_2_), 7.32–7.87 (m, 12H, Ar–H + pyridine H5), 8.10 (s, 1H, CH=N (azomethine protone)), 11.08 (s, 1H, NH, D_2_O exchangeable); ^13^C-NMR (DMSO-*d*_6_): 64.1 (CH_2_), 117.5 (CN), 120.98, 121.01, 121.48, 123.40, 125.21, 127.83, 128.03, 128.99, 132.06, 133.56, 137.98, 138.43, 141.32, 142.65, 144.64, 146.10, 149.01, 149.97, 150.16, 150.63 (23 Ar–C), 157.2 (C=N), 159.4 (CH=N); MS, *m*/*z* (%): 536 [M]^+^ (97), 538 [M + 2]^+^ (64), 540 [M + 4]^+^ (11). Anal. Calcd. for C_27_H_16_Cl_2_F_2_N_4_O_2_ (537.34): C, 60.35; H, 3.00; N, 10.43%; found: C, 60.56 ; H, 3.21; N, 10.65%.

#### 3.2.7. 2-Chloro-6-(2,4-dichlorophenyl)-4-(4-fluorophenyl)nicotinonitrile (**11**)

A mixture of compound **2** (3.6 g, 0.01 mol) and phosphorus oxychloride (4.6 mL, 0.03 mol) was refluxed for 8 h. The reaction mixture was poured into crushed ice and the separated solid was filtered, dried and recrystallized from dioxane to give compound 11. Yield 62%; m.p. 181–182 °C; IR (KBr) ν_max_ in cm^−1^: 2223 (CN); ^1^H-NMR (DMSO-*d*_6_): 6.80 (s, 1H, pyridine H5), 7.62–8.01 (m, 7H, Ar–H); ^13^C-NMR (DMSO-*d*_6_): 119.2 (CN), 122.31, 125.86, 127.90, 128.54, 131.98, 133.32, 138.32, 139.09, 144.89, 145.07, 148.48 (16 Ar–C), 158.7 (C=N); MS, *m*/*z* (%): 376 [M]^+^ (62), 378 [M + 2]^+^ (60), 380 [M + 4]^+^ (18); 382 [M + 6]^+^ (2). Anal. Calcd. for C_18_H_8_Cl_3_FN_2_ (377.63): C, 57.25; H, 2.14; N, 7.42%; found: C, 57.10; H, 2.43; N, 7.65%.

#### 3.2.8. General procedure for the synthesis of 2-(benzylamino)-6-(2,4-dichlorophenyl)-4-(4-fluoro-phenyl)-nicotinonitrile (**12**), 2-(benzo[*d*][1,3]dioxol-5-ylmethylamino)-6-(2,4-dichlorophenyl)-4-(4-fluorophenyl)nicotinonitrile (**13**), 6-(2,4-dichlorophenyl)-4-(4-fluorophenyl)-2-(1-phenylethyl-amino)nicotinonitrile (**14**), and 6-(2,4-dichlorophenyl)-4-(4-fluorophenyl)-2-(2-substituted-1-yl)nicotinonitriles **15a**,**b**

A mixture of chloropyridine **11** (3.8 g, 0.01 mol) and the appropriate amine, namely benzyl-amine, piperonylamine, 1-phenylethylamine, 2-methylpiperidine and/or morpholine (0.01 mol) in absolute ethanol (30 mL) was refluxed for 6–12 h. The reaction mixture was poured onto ice/cold water, filtered, washed with petroleum ether 60–80 and finally crystallized from ethanol to give the desired derivatives **12**–**15a**,**b**, respectively.

*2-(Benzylamino)-6-(2,4-dichlorophenyl)-4-(4-fluorophenyl)nicotinonitrile* (**12**): Yield 48%; m.p. 158–159 °C; IR (KBr) ν_max_ in cm^−1^: 3218 (NH), 2220 (CN); ^1^H-NMR (DMSO-*d*_6_): 4.97 (s, 2H, CH_2_), 6.98 (br s, 1H, NH, D_2_O exchangeable), 7.62–8.31 (m, 13H, Ar–H + pyridine H5); ^13^C-NMR (DMSO-*d*_6_): 51.45 (CH_2_), 118.7 (CN), 120.01, 120.96, 122.73, 124.65, 128.02, 129.90, 131.45, 133.63, 134.06, 136.81, 139.43, 141.45, 144.09, 146.12, 147.48, 148.18, 149.74, 150.23, 153.42 (22 Ar–C), 159.2 (C=N); MS, *m*/*z* (%): 447 [M]^+^ (30), 449 [M + 2]^+^ (17), 451 [M + 4]^+^ (3). Anal. Calcd. for C_25_H_16_Cl_2_FN_3_ (448.32): C, 66.98; H, 3.60; N, 9.37%; found: C, 67.10 ; H, 3.40; N, 9.55%.

*2-(Benzo[d][1,3]dioxol-5-ylmethylamino)-6-(2,4-dichlorophenyl)-4-(4-fluorophenyl)nicotinonitrile* (**13**): Yield 59%; m.p. 149–151 °C; IR (KBr) ν_max_ in cm^−1^: 3229 (NH), 2218 (CN); ^1^H-NMR (DMSO-*d*_6_): 4.85 (s, 2H, CH_2_), 6.21 (s, 2H, CH_2_), 7.02 (br s, 1H, NH, D_2_O exchangeable), 7.68–8.22 (m, 11H, Ar–H + pyridine H5); ^13^C-NMR (DMSO-*d*_6_): 49.5, 88.6 (2CH_2_), 117.2 (CN), 121.72, 122.98, 124.61, 128.03, 131.43, 133.92, 134.98, 137.38, 138.51, 142.26, 143.13, 144.06, 144.97, 148.26, 149.49 (22 Ar–C), 158.7 (C=N); MS, *m*/*z* (%): 491 [M]^+^ (19), 493 [M + 2]^+^ (13), 495 [M + 4]^+^ (2). Anal. Calcd. for C_26_H_16_Cl_2_FN_3_O_2_ (492.33): C, 63.43; H, 3.28; N, 8.53%; found: C, 63.66 ; H, 3.47; N, 8.41%.

*6-(2,4-Dichlorophenyl)-4-(4-fluorophenyl)-2-(phenylethylamino)nicotinonitrile* (**14**): Yield 32%; m.p. 142–143 °C; IR (KBr) ν_max_ in cm^−1^: 3203 (NH), 2228 (CN); ^1^H-NMR (DMSO-*d*_6_): 2.93 (m, 2H, CH_2_), 3.39 (m, 2H, CH_2_), 7.32 (br s, 1H, NH, D_2_O exchangeable), 7.76–8.36 (m, 13H, Ar–H + pyridine H5); ^13^C-NMR (DMSO-*d*_6_): 39.1, 46.9 (2CH_2_), 119.1 (CN), 122.01, 122.86, 123.84, 124.62, 127.97, 128.12, 131.82, 133.27, 136.46, 139.65, 141.54, 144.87, 146.32, 147.07, 148.86, 149.41 (22 Ar–C), 159.0 (C=N); MS, *m*/*z* (%): 461 [M]^+^ (32), 463 [M + 2]^+^ (23), 465 [M + 4]^+^ (3). Anal. Calcd. for C_26_H_18_Cl_2_FN_3_ (462.35): C, 67.54; H, 3.92; N, 9.09%; found: C, 67.69 ; H, 3.71; N, 8.89%.

*6-(2,4-Dichlorophenyl)-4-(4-fluorophenyl)-2-(2-methylpiperidin-1-yl)nicotinonitrile* (**15a**): Yield 47%; m.p. 98–100 °C; IR (KBr) ν_max_ in cm^−1^: 2219 (CN); ^1^H-NMR (DMSO-*d*_6_): 1.36 (s, 3H, CH_3_), 1.59–1.73 (m, 6H, 3CH_2_-piperidine protons), 2.78 (m, 3H, (CH + CH_2_) piperidine protons), 7.48–8.01 (m, 8H, Ar–H + pyridine H5); ^13^C-NMR (DMSO-*d*_6_): 18.6 (CH_3_), 22.4, 25.2, 36.9, 49.3 (4CH_2_), 56.7 (CH), 119.7 (CN), 120.76, 121.20, 125.85, 128.32, 129.04, 132.85, 133.25, 137.87, 138.13, 139.08, 141.24, 143.79, 143.9 (16 Ar–C), 157.9 (C=N). Anal. Calcd. for C_24_H_20_Cl_2_FN_3_ (440.34): C, 65.46; H, 4.58; N, 9.54%; found: C, 65.70 ; H, 4.32; N, 9.39%.

*6-(2,4-Dichlorophenyl)-4-(4-fluorophenyl)-2-morpholinonicotinonitrile* (**15b**): Yield 32%; m.p. 112–114 °C; IR (KBr) ν_max_ in cm^−1^: 2227 (CN); ^1^H-NMR (DMSO-*d*_6_): 3.31 (m, 4H, 2N-C*H*_2_), 3.72 (m, 4H, 2O-C*H*_2_), 7.52–7.78 (m, 8H, Ar–H + pyridine H5); ^13^C-NMR (DMSO-*d*_6_): 47.9, 49.1 (2N-*C*H_2_), 64.10, 65.9 (2O–*C*H_2_), 119.1 (CN), 120.46, 122.04, 122.83, 126.23, 127.85, 129.27, 131.47, 133.56, 134.26, 138.93, 139.08, 142.86, 144.75, 146.12, 146.91, 147.15 (16 Ar–C), 158.9 (C=N). Anal. Calcd. for C_22_H_16_Cl_2_FN_3_O (428.29): C, 61.70; H, 3.77; N, 9.81%; found: C, 61.88 ; H, 3.42; N, 9.69%.

#### 3.2.9. 2-[3-Cyano-6-(2,4-dichlorophenyl)-4-(4-fluorophenyl)pyridin-2-yl]malononitrile (**16**)

To a solution of compound **11** (3.8 g, 0.01 mol) in absolute ethanol (30 mL), triethylamine (1 mL), and malononitrile (0.7 g, 0.01 mol) were added. The reaction mixture was refluxed for 6 h, then left to cool to room temperature, poured into cold water and neutralized with diluted hydrochloric acid to complete precipitation. The solid obtained was filtered, washed with water, dried and recrystallized from ethanol to give compound **16**. Yield 39%; m.p. 99–101 °C; IR (KBr) ν_max_ in cm^−1^: 2218, 2225 (CN); ^1^H-NMR (DMSO-*d*_6_): 5.02 (s, 1H, CH), 7.35–8.13 (m, 8H, Ar–H + pyridine H5); ^13^C-NMR (DMSO-*d*_6_): 32.8 (CH), 117.8, 119.5 (3CN), 120.76, 122.45, 123.26, 124.01, 124.81, 128.34, 129.27, 132.23, 133.84, 137.37, 139.06, 142.29, 144.52, 145.14, 146.17, 147.91 (16 Ar–C), 159.3 (C=N); MS, *m*/*z* (%): 406 [M]^+^ (96), 408 [M + 2]^+^ (62), 410 [M + 4]^+^ (11). Anal. Calcd. for C_21_H_9_Cl_2_FN_4_ (407.23): C, 61.94; H, 2.23; N, 13.76%; found: C, 62.05; H, 2.39; N, 13.63%. 

#### 3.2.10. 6-(2,4-Dichlorophenyl)-4-(4-fluorophenyl)-2-hydrazinylnicotinonitrile (**17**)

A mixture of the chloropyridine derivative **11** (3.8 g, 0.01 mol) and hydrazine hydrate (98%, 2 mL, 0.04 mol) in ethanol (20 mL) was stirred under reflux for 6 h. The formed precipitate was filtered, dried and recrystallized from methanol to give the hydrazinyl derivative **17**. Yield 85%; m.p. 223–224 °C; IR (KBr) ν_max_ in cm^−1^: 3440, 3320, 3150 (NH_2_, NH), 2218 (CN); ^1^H-NMR (DMSO-*d*_6_): 5.40 (s, 2H, NH_2_, D_2_O exchangeable), 7.39–8.21 (m, 8H, Ar–H + pyridine H5), 9.40 (s, 1H, NH, D_2_O exchangeable); ^13^C-NMR (DMSO-*d*_6_): 119.5 (CN), 121.09, 122.58, 123.71, 125.35, 129.34, 132.98, 134.46, 138.47, 139.07, 142.32, 144.35, 145.07, 147.43, 148.54, 149.76, 150.12 (16 Ar–C), 158.9 (C=N); MS, *m*/*z* (%): 372 [M]^+^ (39), 374 [M + 2]^+^ (21), 346 [M + 4]^+^ (4). Anal. Calcd. for C_18_H_11_Cl_2_FN_4_ (373.21): C, 57.93; H, 2.97; N, 15.01%; found: C, 58.23; H, 2.80; N, 14.89%.

#### 3.2.11. General procedure for the synthesis of 6-(2,4-dichlorophenyl)-4-(4-fluorophenyl)-2-(3-methyl-5-oxo-4,5-dihydro-1*H*-pyrazol-1-yl)nicotinonitrile (**18**) and 6-(2,4-dichlorophenyl)-2-(3,5-dimethyl-1*H*-pyrazol-1-yl)-4-(4-fluorophenyl)nicotinonitrile (**19**)

A mixture of compound **17** (3.7 g, 0.01 mol) and ethyl acetoacetate or acetylacetone (0.01 mol) in acetic acid (15 mL) was refluxed for 8 h. The solid formed after cooling was filtered, dried and recrystallized from ethanol to give compounds **18**, and **19** respectively.

*6-(2,4-Dichlorophenyl)-4-(4-fluorophenyl)-2-(3-methyl-5-oxo-4,5-dihydro-1H-pyrazol-1-yl) nicotinonitrile* (**18**): Yield 40%; m.p. 246–248 °C; IR (KBr) ν_max_ in cm^−1^: 2227 (CN), 1701 (C=O); ^1^H-NMR (DMSO-*d*_6_): 1.94 (s, 3H, CH_3_), 2.26 (s, 2H, CH_2_), 7.52–7.78 (m, 8H, Ar–H + pyridine H5); ^13^C-NMR (DMSO-*d*_6_): 19.3 (CH_3_), 41.5 (CH_2_), 118.3 (CN), 122.62, 123.42, 126.81, 129.17, 131.11, 133.91, 136.23, 137.05, 139.54, 14.13, 142.53, 143.06, 143.94 (16 Ar–C), 158.4, 159.3 (2C=N), 166.5 (C=O); MS, *m*/*z* (%): 438 [M]^+^ (16), 440 [M + 2]^+^ (11), 442 [M + 4]^+^ (2). Anal. Calcd. for C_22_H_13_Cl_2_FN_4_O (439.24): C, 60.15; H, 2.98; N, 12.75%; found: C, 60.25; H, 3.12; N, 12.54%.

*6-(2,4-Dichlorophenyl)-2-(3,5-dimethyl-1H-pyrazol-1-yl)-4-(4-fluorophenyl)nicotinonitrile* (**19**): Yield 32%; m.p. 287–289 °C; IR (KBr) ν_max_ in cm^−1^: 2210 (CN); ^1^H-NMR (DMSO-*d*_6_): 2.31 (s, 3H, CH_3_), 2.45 (s, 3H, CH_3_), 6.15 (s, 1H, CH-pyrazole), 7.46–8.09 (m, 8H, Ar–H + pyridine H5); ^13^C-NMR (DMSO-*d*_6_): 18.4, 19.3 (2CH_3_), 108.4 (CH-pyrazole), 117.9 (CN), 122.94, 123.27, 124.18, 128.97, 129.46, 132.74, 133.24, 136.93, 138.08, 139.60, 143.84, 144.94, 148.23, 148.86 (17 Ar–C), 158.3, 159.5 (2C=N); MS, *m*/*z* (%): 436 [M]^+^ (12), 438 [M + 2]^+^ (8), 440 [M + 4]^+^ (1). Anal. Calcd. for C_23_H_15_Cl_2_FN_4_ (437.30): C, 63.17; H, 3.46; N, 12.81%; found: C, 62.98; H, 3.17; N, 12.61%.

#### 3.2.12. 6-(2,4-Dichlorophenyl)-4-(4-fluorophenyl)-2-[2-(2-oxoindolin-3-ylidene)hydrazinyl]nicotinonitrile (**20**)

A mixture of the compound **17** (3.7 g, 1 mmol) and isatin (1.5 g, 1 mmol) in ethanol (25 mL) containing 3 drops of acetic acid was refluxed for 2 h, then left overnight at room temperature. The formed precipitate was filtered, dried and recrystallized from benzene to give **20**. Yield 79%; m.p. 268–269 °C; IR (KBr) ν_max_ in cm^−1^: 3289, 3150 (2NH), 1723 (C=O), 2227 (CN); ^1^H-NMR (DMSO-*d*_6_): 6.98 (br s, 1H, NH, D_2_O exchangeable), 7.22–8.24 (m, 12H, Ar–H + pyridine H5), 10.02 (s, 1H, NH, D_2_O exchangeable); ^13^C-NMR (DMSO-*d*_6_): 120.01 (CN), 120.42, 121.07, 122.86, 123.07, 124.52, 129.05, 129.96, 131.46, 133.63, 135.64, 137,93, 139.75, 141.25, 143.86, 144.52, 145.28, 147.94, 148.6 149.25 (22 Ar–C), 157.9, 158.3 (2C=N), 167.23 (C=O). MS, *m*/*z* (%): 501 [M]^+^ (85), 503 [M + 2]^+^ (60), 505 [M + 4]^+^ (9). Anal. Calcd. for C_26_H_14_Cl_2_FN_5_O (502.33): C, 62.17; H, 2.81; N, 13.94%; found: C, 61.97; H, 2.60; N, 14.19%.

#### 3.2.13. General procedure for the synthesis of 6-(2,4-dichlorophenyl)-4-(4-fluorophenyl)-2-[2-(2-substiutedbenzylidene)hydrazinyl]nicotinonitriles (**1a**–**e**)

A mixture of compound **17** (3.7 g, 0.01 mol), an appropriate aromatic aldehyde namely 2-bromo-benzaldhyde, 4-nitrobenzaldhyde, 2-methoxybenzaldhyde, 3,4-dimethoxybenzaldhyde and/or 1-naphthaldehyde (0.01 mol) in ethanol (20 mL) containing 3 drops of acetic acid was refluxed for 6–8 h. The precipitate formed after cooling was filtered, dried and recrystallized to give compounds **21a**–**e**, respectively.

*2-[2-(2-Bromobenzylidene)hydrazinyl]-6-(2,4-dichlorophenyl)-4-(4-fluorophenyl)nicotinonitrile* (**21a**): Yield 41%; m.p. 226–228 °C; IR (KBr) ν_max_ in cm^−1^: 3299 (NH), 2217 (CN); ^1^H-NMR (DMSO-*d*_6_): 7.26–8.19 (m, 12H, Ar–H + pyridine H5), 8.32 (s, 1H, CH=N azomethine proton), 10.13 (s, 1H, NH, D_2_O exchangeable); ^13^C-NMR (DMSO-*d*_6_): 118.6 (CN), 121.83, 122.94, 123.10, 123.86, 127.39, 128.93, 129.27, 132.81, 135.23, 139.38, 141.04, 142.50, 144.52, 148.01, 148.87, 149.08, 150.65, 151.31 (22 Ar–C), 158.3 (C=N), 161.3 (CH=N); MS, *m*/*z* (%): 538 [M]^+^ (14), 540 [M + 2]^+^ (10), 542 [M + 4]^+^ (1). Anal. Calcd. for C_25_H_14_BrCl_2_FN_4_ (540.21): C, 55.58; H, 2.61; N, 10.37%; found: C, 55.68; H, 2.79; N, 10.51%.

*6-(2,4-Dichlorophenyl)-4-(4-fluorophenyl)-2-[2-(4-nitrobenzylidene)hydrazinyl]nicotinonitrile* (**21b**): Yield 23%; m.p. 281–283 °C; IR (KBr) ν_max_ in cm^−1^: 3253 (NH), 2223 (CN); ^1^H-NMR (DMSO-*d*_6_): 7.29–8.14 (m, 12H, Ar–H + pyridine H5), 8.31 (s, 1H, CH=N (azomethine protone)), 10.08 (s, 1H, NH, D_2_O exchangeable); ^13^C-NMR (DMSO-*d*_6_): 119.0 (CN), 121.52, 122.61, 123.48, 127.85, 128.03, 129.53, 130.21, 133.25, 134.56, 136.87, 137.04, 139.08, 139.96, 142.19, 143.15, 144.08, 148.60, 149.31 (22 Ar–C), 158.7 (C=N), 160.8 (CH=N); MS, *m*/*z* (%): 505 [M]^+^ (60), 507 [M + 2]^+^ (39), 509 [M + 4]^+^ (7). Anal. Calcd. for C_25_H_14_Cl_2_FN_5_O_2_ (506.32): C, 59.30; H, 2.79; N, 13.83%; found: C, 59.58; H, 2.63; N, 13.51%.

*6-(2,4-Dichlorophenyl)-4-(4-fluorophenyl)-2-[2-(2-methoxybenzylidene)hydrazinyl] nicotinonitrile* (**21c**): Yield 34%; mp over 300 °C; IR (KBr) ν_max_ in cm^−1^: 3258 (NH), 2218 (CN); ^1^H-NMR (DMSO-*d*_6_): 3.39 (s, 3H, OCH_3_), 7.49–8.09 (m, 12H, Ar–H + pyridine H5), 8.33 (s, 1H, CH=N (azomethine protone)), 10.16 (s, 1H, NH, D_2_O exchangeable); ^13^C-NMR (DMSO-*d*_6_): 51.9 (OCH_3_), 118.7 (CN), 121.09, 122.58, 123.71, 125.35, 129.34, 132.98, 134.46, 137.94, 138.47, 139.07, 142.32, 144.35, 145.07, 147.43, 148.54, 149.76, 150.12, 151.72 (22 Ar–C), 158.1 (C=N), 159.9 (CH=N). Anal. Calcd. for C_26_H_17_Cl_2_FN_4_O (491.34): C, 63.56; H, 3.49; N, 11.40%; found: C, 63.78; H, 3.26; N, 11.57%.

*6-(2,4-Dichlorophenyl)-2-[2-(3,4-dimethoxybenzylidene)hydrazinyl]-4-(4-fluorophenyl) nicotinonitrile* (**21d**): Yield 31%; m.p. 278–280 °C; IR (KBr) ν_max_ in cm^−1^: 3294 (NH), 2220 (CN); ^1^H-NMR (DMSO-*d*_6_): 3.39 (2s, 6H, 2OCH_3_), 7.36–8.10 (m, 11H, Ar–H + pyridine H5), 8.31 (s, 1H, CH=N (azomethine protone)), 10.23 (s, 1H, NH, D_2_O exchangeable); ^13^C-NMR (DMSO-*d*_6_): 55.6, 56.01 (2OCH_3_), 119.3 (CN), 120.46, 121.08, 122.85, 123.05, 127.30, 128.74, 129.76, 130.64, 133.73, 134.05, 138.12, 138.98, 140.16, 143.54, 144.35, 145.97, 147.46, 148.02, 149.08 (22 Ar–C), 158.7 (C=N), 161.4 (CH=N). Anal. Calcd. for C_27_H_19_Cl_2_FN_4_O_2_ (521.37): C, 62.20; H, 3.67; N, 10.75%; found: C, 62.38; H, 3.43; N, 10.53%.

*6-(2,4-Dichlorophenyl)-4-(4-fluorophenyl)-2-[2-(naphthalen-1-ylmethylene)hydrazinyl] nicotinonitrile* (**21e**): Yield 24%; m.p. 296–298 °C; IR (KBr) ν_max_ in cm^−1^: 3282 (NH), 2213 (CN); ^1^H-NMR (DMSO-*d*_6_): 7.24–8.26 (m, 15H, Ar–H + pyridine H5), 8.32 (s, 1H, CH=N (azomethine protone)), 10.18 (s, 1H, NH, D_2_O exchangeable); ^13^C-NMR (DMSO-*d*_6_): 119.0 (CN), 120.76, 122.45, 123.26, 124.01, 124.81, 128.34, 129.27, 132.23, 133.84, 137.37, 139.06, 142.29, 144.52, 145.14, 146.17, 147.91, 148.96, 149.24, 149,99, 150.65, 151.34, 152.61 (26 Ar–C), 158.3 (C=N), 161.0 (CH=N). Anal. Calcd. for C_29_H_17_Cl_2_FN_4_ (511.38): C, 68.11; H, 3.35; N, 10.96%; found: C, 67.96; H, 3.49; N, 11.06%.

### 3.3. Anticancer Activity

#### 3.3.1. Cell Cultures

The newly synthesized compounds were evaluated *in vitro* against three human cancer cell lines; which are MCF-7 (breast adenocarcinoma), NCI-H460 (non-small cell lung cancer) and SF-268 (CNS cancer), and WI 38 (normal fibroblast cells) were used in this study. MCF-7 was obtained from the European Collection of Cell Cultures (ECACC, Salisbury, UK) but NCI-H460, SF-268 and WI 38 were kindly provided by the National Cancer Institute (NCI, Cairo, Egypt). They grow as monolayers routinely maintained in RPMI-1640 medium supplemented with 5% heat inactivated fetal bovine serum (FBS), 2 mM glutamine and antibiotics (penicillin 100 U/mL, streptomycin 100 µg/mL), at 37 °C in a humidified atmosphere containing 5% CO_2_. Exponentially growing cells were obtained by plating 1.5 × 105 cells/mL for MCF-7 and SF-268, and 0.75 × 104 cells/mL for NCI-H460 followed by 24 h of incubation. The effect of the vehicle solvent (DMSO) on the growth of these cell lines was evaluated in all experiments by exposing untreated control cells to the maximum concentration (0.5%) of DMSO used in each assay.

#### 3.3.2. Cancer Cell Growth Assay

The effect of compounds on the *in vitro* growth of human tumor cell lines were evaluated according to the procedure adopted by the National Cancer Institute (NCI, Austin, TX, USA) in the “*In vitro* Anticancer Drug Discovery Screen” that uses the protein-binding dye sulforhodamine B (SRB) to assess cell growth [35]. In the assay protocol, all cells were incubated at 37 °C under humidified atmosphere containing 5% CO_2_. Briefly, exponentially cells growing in 96-well plates were then exposed for 48 h to five serial concentrations of each compound, starting from a maximum concentration of 150 μg/mL. Following this exposure period, adherent cells were fixed, washed and stained. The bound stain was solubilized and the absorbance was measured at 492 nm in a Power Wave XS plate reader (Bio-Tek Instruments Inc., Winston, NC, USA). For each test compound and cell line, a dose response curve was obtained and the inhibitory concentration of 50% (IC_50_), corresponding to the concentration of the compounds that inhibited 50% of the net cell growth was calculated as described elsewhere [37]. Doxorubicin was used as a positive control and tested in the same manner.

## 4. Conclusions

This study focused on the synthesis of a new 1,2-dihydropyridin-3-carbonitrile and nicotinonitrile derivatives as potential anticancer agents. Some of newly synthesized derivatives were examined *in vitro* as cytotoxic agents against three human cancer cell lines. It could be noticed that the ester functionality-bearing derivative **8** and the derivative **16** carrying a malononitrile side chain attached to the parent cyanopyridine nucleus showed the best results, exhibiting the highest inhibitory effects towards the three tumor cell lines, which were higher than that of the reference compound doxorubicin and these compounds were non-cytotoxic towards normal cells (IC_50_ values >100 μg/mL). In addition, compounds **9**, **17** and **20** exhibited high growth inhibitory activity on the various cancer panel cell lines, with weak cytotoxicity on the normal cells.

## References

[1] Zhang J.Y. (2002). Apoptosis-based anticancer drugs. Nat. Rev. Drug Disccov..

[2] Ali A., Fergus K., Wright F.C., Pritchard K.I., Kiss A., Warner E. (2014). The impact of a breast cancer diagnosis in young women on their relationship with their mothers. Breast.

[3] Lam S.W., Jimenez C.R., Boven E. (2014). Breast cancer classification by proteomic technologies: Current state of knowledge. Cancer.

[4] Hassan G.S., Kadry H.H., Abou-Seri S.M., Ali M.M., Mahmoud A.E.E. (2011). Synthesis and *in vitro* cytotoxic activity of novel pyrazolo[3,4-*d*]pyrimidines and related pyrazole hydrazones toward breast adenocarcinoma MCF-7 cell line. Bioorg. Med. Chem..

[5] Taher A.T., Georgey H.H., El-Subbagh H.I. (2012). Novel 1,3,4-heterodiazole analogues: Synthesis and *in vitro* antitumor activity. Eur. J. Med. Chem..

[6] Carmen A.J., Carlos M. (2008). Medicinal Chemistry of Anticancer Drugs.

[7] Borowski E., Bontemps-Gracz M.M., Piwkowska A. (2005). Strategies for overcoming ABC-transporters-mediated multidrug resistance (MDR) of tumor cells. Acta Biochim. Pol..

[8] Avila H.P., Smania E.F., Monache F.D., Smania A. (2008). Structure-activity relationship of antibacterial chalcones. Bioorg. Med. Chem..

[9] Liu Y., Sun X., Yin D., Yuan F. (2013). Syntheses and biological activity of chalcones-imidazole derivatives. Res. Chem. Intermed..

[10] Sortino M., Delgado P., Juarez S., Quiroga J., Abonia R., Insuasty B., Nogueras M., Rodero L., Garibotto F.M., Enriz R.D. (2007). Synthesis and antifungal activity of (*Z*)-5-arylidenerhodanines. Bioorg. Med. Chem..

[11] Lopez S.N., Castelli M.V., Zacchino S.A., Dominguez J.N., Lobo G., Charris-Charris J., Cortes J.C., Ribas J.C., Devia C., Rodriguez A.M. (2001). *In vitro* antifungal evaluation and structure-activity relationships of a new series of chalcone derivatives and synthetic analogues with inhibitory properties against polymers of the fungal cell wall. Bioorg. Med. Chem..

[12] Cheng J.H., Hung C.F., Yang S.C., Wang J.P., Won S.J., Lin C.N. (2008). Synthesis and cytotoxic, anti-inflammatory, and anti-oxidant activities of 2′,5′-dialkoxylchalcones as cancer chemopreventive agents. Bioorg. Med. Chem..

[13] Katsori A.M., Hadjipavlou-Litina D. (2009). Chalcones in cancer: Understanding their role in terms of QSAR. Curr. Med. Chem..

[14] Modzelewska A., Pettit C., Achanta G., Davidson N.E., Huang P., Khan S.R. (2006). Anticancer activities of novel chalcone and bis-chalcone derivatives. Bioorg. Med. Chem..

[15] Abdelhafez O.M., Abdel-Latif N.A., Badria F.A. (2011). DNA, Antiviral activities and cytotoxicity of new furochromone and benzofuran derivatives. Arch. Pharm. Res..

[16] Abdel-Latif N.A. (2005). Synthesis and antidepressant activity of some new coumarin derivatives. Sci. Pharm..

[17] Son J.K., Zhao L.X., Basnet A., Thapa P., Karki R., Na Y., Jahng Y., Jeong T.C., Jeong B.S., Lee C.S. (2008). Synthesis of 2,6-diaryl-substituted pyridines and their antitumor activities. Eur. J. Med. Chem..

[18] Amr A.G., Abdulla M.M. (2006). Anti-inflammatory profile of some synthesized heterocyclic pyridone and pyridine derivatives fused with steroidal structure. Bioorg. Med. Chem..

[19] Hammam A.G., Abdel Hafez N.A., Midura W.H., Mikolajczyk M.Z. (2000). Chemistry of seven-membered heterocycles, VI. Synthesis of novel bicyclic heterocyclic compounds as potential anticancer and anti-HIV agents. Z. Naturforsch..

[20] Kotb E.R., Anwar M.M., Abbas H.A.S., Abd El-Moez S.I. (2013). A concise synthesis and antimicrobial activity of a novel series of naphthylpyridine-3-carbonitrile compounds. Acta Pol. Pharm. Drug Res..

[21] Sayed H.H., Morsy E.M., Flefel E.M. (2010). Synthesis and reactions of some novel nicotinonitrile, thiazolotriazole, and imidazolotriazole derivatives for antioxidant evaluation. Synth. Commun..

[22] Akira M., Aya N., Shigeki I., Motoki T., Kazuo S. (2009). JBIR-54, a new 4-pyridinone derivative isolated from Penicillium daleae Zaleski fE50. J. Antibiot..

[23] Al-Omar M.A., Amr A.E., A.l-Salahi R.A. (2010). Anti-inflamatory, analgesic, anticonvulsant and antiparkinsonian activities of some pyridine derivatives using 2,6-disubstituted isonicotinic acid hydrazides. Archiv. Phaem..

[24] Martin C., Göggel R., dal Piaz V., Vergelli C., Giovannoni P., Ernst M., Uhlig S. (2002). Airway relaxant and anti-inflammatory properties of a PDE4 inhibitor with low affinity for the high-affinity rolipram binding site. Naunyn-Schmiedeberg’s Arch. Pharmacol..

[25] Amr A.E., Sayed H.H., Abdulla M.A. (2005). Synthesis and reactions of some new substituted pyridine and pyrimidine derivatives as analgesic, anticonvulsant and antiparkinsonian agents. Arch. Pharm. Chem. Life Sci..

[26] Al-Abdullah E.S. (2011). Synthesis and anticancer activity of some novel tetralin-6-yl-pyrazoline, 2-thioxopyrimidine, 2-oxopyridine, 2-thioxo-pyridine and 2-iminopyridine derivatives. Molecules.

[27] Abo-Ghalia M., Abdulla M.M.Z., Amr A.E. (2003). Synthesis of some new (N^α^-dipicolinoyl)-*bis*-l-leucyl-dl-norvalyl linear tetra and cyclic octa bridged peptides as new antiinflammatory agents. Z. Naturforsch..

[28] Kotb E.R., El-Hashash M.A., Salama M.A., Kalf H.S., Abdel Wahed N.A.M. (2009). Synthesis and reactions of some novel nicotinonitrile derivatives for anticancer and antimicrobial evaluation. Acta Chim. Slov..

[29] Kumar S., Das S., Dey S., Maity P., Guha M., Choubey V., Panda G., Bandyopadhyay V. (2008). Antiplasmodial activity of [(aryl)arylsulfanylmethyl]pyridine. Antimicrob. Agents Chemother..

[30] Ghosh P.S., Manna K., Banik U., Das M., Sarkar P. (2014). Synthetic strategies and pharmacology of 2-oxo-3-cyanopyridine derivatives: A review. Int. J Pharm. Pharm. Sci..

[31] Abbas H.-A.S., El Sayed W.A., Fathy N.M. (2010). Synthesis and antitumor activity of new dihydropyridine thioglycosides and their corresponding dehydrogenated forms. Eur. J. Med. Chem..

[32] Al-Mutairi M.S., Al-Abdullah E.S., Haiba M.E., Khedr M.A., Zaghary W.A. (2012). Synthesis, molecular docking and preliminary *in vitro* cytotoxic evaluation of some substituted tetrahydronaphthalene (2′,3′,4′,6′-Tetra-*O*-Acetyl-β-d-Gluco-/Galactopyranosyl) derivatives. Molecules.

[33] Kotb E.R., Abbas H.-A.S., Flefel E.M., Sayed H.H., Abdel Wahed N.A.M. (2015). Utility of hantzsch ester in synthesis of some 3,5-bisdihydropyridine derivatives and studying their biological evaluation. J. Heterocycl. Chem..

[34] Sayed H.H., Flefel E.M., Abd El-Fatah A.M., El-Sofany W.I. (2010). Focus on the synthesis and reactions of some new pyridine carbonitrile derivatives as antimicrobial and antioxidant agents. Egypt J. Chem..

[35] Skehan P., Storeng R., Scudiero D., Monks A., McMahon J., Vistica D., Warren J.T., Bokesch H., Kenne S., Boyd M.R. (1990). New colorimetric cytotoxicity assay for anticancer-drug screening. J. Natl. Cancer Inst..

[36] Lee P., Zhang R., Li V., Liu X., Sun R.W.Y., Che C.M., Wong K.K.Y. (2012). Enhancement of anticancer efficacy using modified lipophilic nanoparticle drug encapsulation. Int. J. Nanomed..

[37] Monks A., Scudiero D., Skehan P., Shoemaker R., Paul K., Vistica D., Hose C., Langley J., Cronise P., Vaigro-Wolff A. (1991). Feasibility of a high-flux anticancer drug screen using a diverse panel of cultured human tumor cell lines. J. Natl. Cancer Inst..

